# Gonadotropin-releasing hormone analog therapies for children with central precocious puberty in the United States

**DOI:** 10.3389/fped.2022.968485

**Published:** 2022-10-04

**Authors:** Jadranka Popovic, Mitchell E. Geffner, Alan D. Rogol, Lawrence A. Silverman, Paul B. Kaplowitz, Nelly Mauras, Philip Zeitler, Erica A. Eugster, Karen O. Klein

**Affiliations:** ^1^Department of Pediatric Endocrinology, Pediatric Institute, Allegheny Health Network, Pittsburgh, PA, United States; ^2^Department of Pediatric Endocrinology, Diabetes and Metabolism, The Saban Research Institute, Children's Hospital Los Angeles, Keck School of Medicine of the University of Southern California, Los Angeles, CA, United States; ^3^Department of Pediatric Diabetes and Endocrinology, University of Virginia, Charlottesville, VA, United States; ^4^Department of Pediatric Endocrinology, Goryeb Children's Hospital Atlantic Health, Morristown, NJ, United States; ^5^Department of Endocrinology, Children's National Hospital, Washington, DC, United States; ^6^Department of Pediatrics, Nemours Children's Health System, Jacksonville, FL, United States; ^7^Department of Pediatric Endocrinology, University of Colorado School of Medicine, Aurora, CO, United States; ^8^Department of Pediatric Endocrinology, Riley Hospital for Children at Indiana University Health, Indianapolis, IN, United States; ^9^Department of Pediatrics, Rady Children's Hospital, University of California, San Diego, San Diego, CA, United States

**Keywords:** central precocious puberty (CPP), gonadotropin-releasing hormone (GnRH) agonists, leuprolide acetate, triptorelin pamoate, histrelin acetate

## Abstract

Gonadotropin-releasing hormone agonists (GnRHa's) are the standard treatment for children with central precocious puberty (CPP). We aim to present data on available GnRHa options with an easy-to-review table and discuss factors that influence treatment selection. Five GnRHa's are currently FDA-approved and prescribed in the US and published data suggest similar safety and efficacy profiles over the first year of treatment. One- and 3-month intramuscular (IM) leuprolide acetate (LA) have long-term safety and efficacy data and allow for flexible dosing. Six-month IM triptorelin pamoate offers a longer duration of treatment, but without long-term efficacy and outcome data. Six-month subcutaneous (SQ) LA combines a SQ route of injection and long duration of action but lacks long-term efficacy and outcome data. The 12-month SQ histrelin acetate implant avoids injections and offers the longest duration of action, but requires a minor surgical procedure with local or general anesthesia. Factors in treatment selection include route of administration, needle size, injection volume, duration of action, and cost. The current GnRHa landscape provides options with varying benefits and risks, allowing physicians and caregivers to select the most appropriate therapy based on the specific needs and concerns of the child and the caregiver. Agents have different advantages and disadvantages for use, with no one agent displaying superiority.

## Introduction

Pubertal maturation typically starts between ages 8–13 years in girls and 9–14 years in boys (1). Children with central precocious puberty (CPP) exhibit puberty earlier as a result of premature activation of the hypothalamic-pituitary-gonadal (HPG) axis (2). A significant long-term consequence of untreated CPP is accelerated skeletal maturation, which can result in premature epiphyseal fusion and, consequently, short adult stature and/or failure to reach genetic target height range (3). Effective CPP treatment can increase adult height and improve the likelihood of achieving one's genetic target height range (4). However, some children reach their target height without treatment, so initiation of CPP treatment is not required in all children presenting with early puberty (5). The short-term goal of treating children with CPP encompasses stabilization or reversal of pubertal maturation, thus potentially reducing social anxiety by aligning the child's pubertal development with that of their peers (6–8). Boys with early-onset puberty may have behavioral difficulties and poor psychological adjustment (9), and girls may experience increased stress from early breast development and onset of menses (8). Indeed, girls who experience early menarche are also at risk of depressive symptoms and anti-social behaviors from adolescence into early-middle adulthood (10), as well as lower quality of life (11, 12).

**Figure 1:**
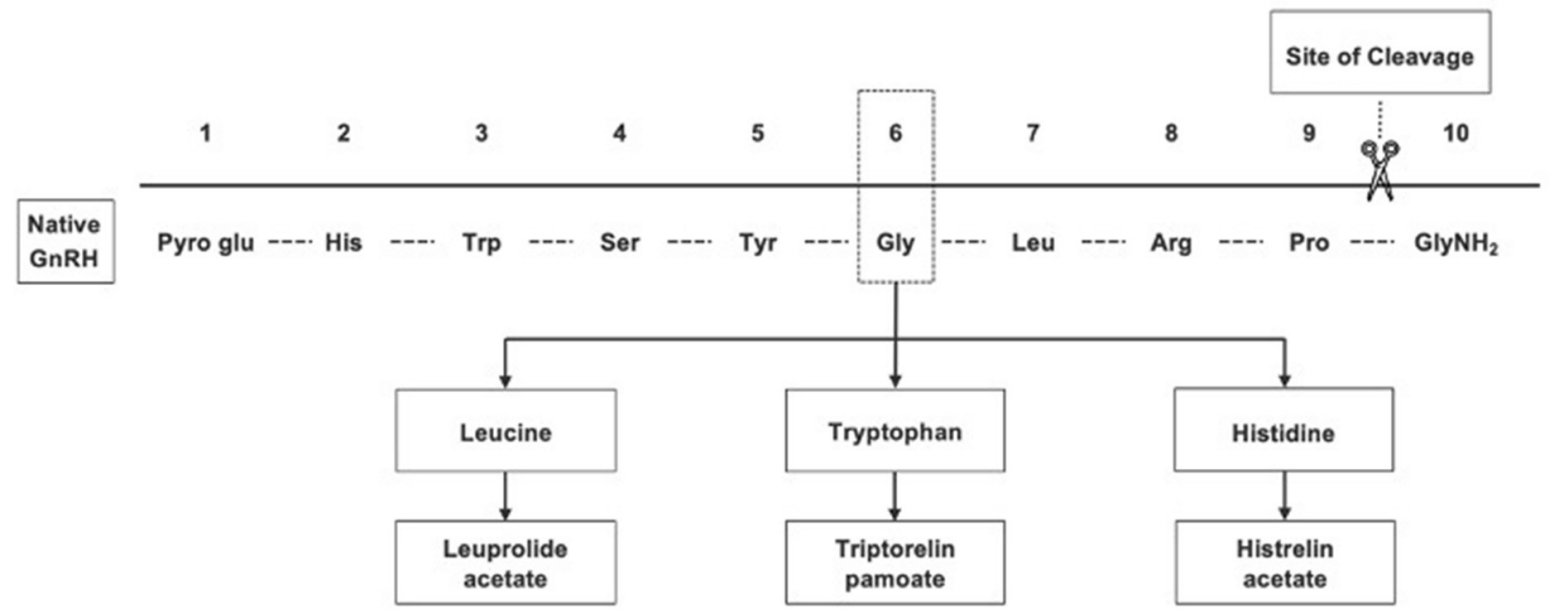
Gonadotropin-releasing hormone agonists, which were synthesized from native GnRH, have greater potency and longer half-lives than native GnRH.

Gonadotropin-releasing hormone (GnRH) agonists (GnRHa's) are standard treatment for CPP (8). The most commonly used therapies in the US are 1- or 3-month intramuscular (IM) leuprolide acetate (LA) (LUPRON DEPOT-PED^®^), 6-month IM triptorelin pamoate (TRIPTODUR^®^), 6-month subcutaneous (SQ) LA (FENSOLVI^®^), and the 12-month histrelin acetate SQ implant (SUPPRELIN^®^) (13–17). The structural modifications to native GnRH that formed each of these GnRHa are shown in Figure 1. Daily SQ LA and twice-daily intranasal GnRHa therapies have previously been used. Nafarelin acetate (SYNAREL^®^) is still available, but concerns about adherence have limited its use, so details are not included here (18).

A recent review by an international group of experts highlighted trends in the care of children with CPP (e.g., fewer GnRH/GnRHa stimulation tests, a shift to longer-acting pharmacological agents, and giving long-acting injections subcutaneously rather than intramuscularly), as well as some future recommendations (e.g., confirming treatment failure on clinical grounds alone and the need for long-term outcome studies) (19). These observations and recommendations established the need for continued therapeutic innovation. We reviewed factors that may affect child and caregiver treatment decisions, including efficacy, route of administration, needle size, injection volume, duration of action, and cost. Treatments for children with CPP are frequently administered for several years (20), and the impact of ongoing treatment on quality of life is particularly important in a pediatric population (21). For example, a very young child will require many more injections or implant exchanges over time, so age may also affect the decision to treat.

In this review, we aimed to provide side-by-side information regarding available treatments in the US for children with CPP, including efficacy and safety data, along with other relevant factors affecting treatment experience for children and clinicians that may help when selecting the most appropriate therapy. Comparisons across studies must take into consideration confounding factors, such as differences in the years when studies were undertaken, populations, and hormone assays (Table 1). Pivotal trial data (defined as the study from which FDA approval was obtained) are summarized. For ease of review, data are organized into a table (Table 2) that includes efficacy and other relevant characteristics. This review also addresses how healthcare providers may apply recently published treatment guidance from professional societies to their clinical practice. We focus on agents available in the USA, as these same analogs are used globally. However, countries may have different preparations and doses available, and listing them all is beyond the scope of this review.

**Table 1:** Trial characteristics of CPP therapies (approved in US) in pivotal trials.

**Drug name**	**Duration of action (months)**	**Dose (mg)**	**Years of study**	**Individual patient trial duration (months)**	**Number of patients**
LUPRON DEPOT-PED (13, 22–24)	1	7.5	1991–2009	48	55
	3	11.25, 30	2008–2010	6	84
TRIPTODUR (15)	6	22.5	2012–2014	12	44
FENSOLVI (16)	6	45	2015–2018	12	64
SUPPRELIN (17, 25)	12	50	2004–2012	12	36

Pivotal trials for CPP therapies were conducted during different decades and had varying durations.

CPP, central precocious puberty; NR, not reported.

**Table 2:** Biochemical and clinical pubertal suppression from pivotal trials of CPP therapies (approved in US).

	**LUPRON DEPOT PED** **(****22****–****24****)**			**TRIPTODUR (26)**	**FENSOLVI (27)**	**SUPPRELIN (28)**
Duration of action (months)	1	3 (11.25 mg)	3 (30 mg)	6	6	12
LH suppression^*^						
Primary outcome: % below peak-stimulated LH threshold (% of patients)	LH <1.75 IU/L: 91^a^	LH <4 IU/L: 78^b^	LH <4 IU/L: 95^b^	LH ≤ 5 IU/L: 93^c^	LH <4 IU/L: 87^a^	LH <4 IU/L: 100^d^
Mean peak-stimulated LH (IU/L)	0.8^a^	≤ 2.5^e^	≤ 2.5^e^	2.0–4.2^d^	3.0^a^	0.8^f^
Mean random LH (IU/L)	NR	NR	NR	0.4–0.7^d^	0.6^a^	0.4^f^
GnRH receptor stimulating agent	Factrel 100 mcg IV	SQ leuprolide acetate 20 mcg/kg	SQ leuprolide acetate 20 mcg/kg	SQ leuprolide acetate 20 mcg/kg	SQ leuprolide acetate 20 mcg/kg or 500 mcg aqueous leuprolide acetate	Leuprolide acetate 20 mcg/kg IV
IU version	1	4	4	5	5	ND
LH assay	DELFIA^TM^ assay	Immuno-chemiluminometric assay	Immuno-chemiluminometric assay	Fluoro-immunometric assays with auto DELFIA^TM^ TRFIA reagents	ECLIA assay	Immuno-chemiluminescent assay
Assay LLOD (IU/L)	0.15	0.02	0.02	0.01	0.10	0.02
Estradiol (E2) suppression^**^						
Prepubertal E2 definition (pg/mL)	NR	<20	<20	<20	<20	<20
Mean E2 (pg/mL)	5.0^g^	1.8^h^	2.8^h^	NR	10.6^i^	5.6^j^
E2 <20 pg/mL (% patients)	99.2^k^	100^l^	100^l^	79.5–92.3^d^	97^i^	79^m^
E2 <10 pg/mL (% patients)	99.2^k^	NR	NR	NR	98^n^	79^m^
Proportion not achieving E2 <20 pg/mL % (*n*)	NR	0% (0 of 39)^l^	0% (0 of 37)^l^	7.7–20.5% (3–8 of 39)^d^	3% (2 of 60)^i^	21%^o^
E2 assay	Radio- immunoassay	HPLC with tandem mass spectrometry	HPLC with tandem mass spectrometry	Radio-immunoassay	LC-MS/MS	Radio-immunoassay and LC-MS/MS
E2 Assay LLOD (pg/mL)	5.0	1.0	1.0	0.9	10.0	5.0
**Testosterone (T) suppression**						
Prepubertal T definition (ng/dL)	<10	<30	<30	<30	<28.4	<30
Mean T (ng/dL)	17.8^p^	11.5^h^	14.4^h^	NR	15.9^a^	NR
T <30 ng/dL (% Patients)	NR	67^l^	100^l^	80–100^d^	50–100^d^	100^d^
Proportion not achieving T <30 ng/dL % (*n*)	NR	33% (1 of 3)^l^	0% (0 of 5)^l^	0–20% (0–1 of 5)^d^	0–50% (0–1 of 2)^d^	0% (0 of 3)^d^
T assay	Radio -immunoassay	HPLC with tandem mass spectrometry	HPLC with tandem mass spectrometry	LC-MS/MS	Chemi -luminescent microparticle immunoassay	Radio -immunoassay
T Assay LLOD (ng/dL)	10.0	3.0	3.0	1.4	11.5	3.0
Number of boys	6	3	5	5	2	3
**Clinical pubertal suppression**						
Baseline BA/CA	1.5	NR	NR	1.4	NR	1.4
BA/CA	0.7^q^	NR	NR	1.3^r^	NR	1.2^s^
Growth (cm/yr)	5.0–6.0^t^	5.9^u^	6.7^u^	6.8^v^	6.0^w^	NR
Pubertal staging	Stabilized or regressed^x^	Stabilized or regressed^y^	Stabilized or regressed^z^	Stabilized or regressed^aa^	Stabilized or regressed^ab^	Minimal maturation^ac^

Suppression of luteinizing hormone to prepubertal concentrations is the primary efficacy endpoint for CPP therapies. These data are similar across CPP therapies. Ninety-seven percentage or more girls achieved E2 suppression to prepubertal levels (<20 pg/mL) in pivotal trials for IM LA, SQ LA, and SQ histrelin implant. In pivotal trials that reported mean T data, all boys achieved suppression to prepubertal levels (<30 ng/dL). Stabilization or regression of pubertal progression was observed in all pivotal trials.

CPP, Central Precocious Puberty; LH, Luteinizing hormone; NR, Not reported; ND, Not determined; IU, International unit; IU Version, which reference preparation the definition of “1 IU” was based on, as the WHO regularly updates the definition when the reference preparation is depleted; LLOD, Lower limit of detection; ECLIA, Electrochemiluminescence immunoassay; E2, Estradiol; HPLC, High-Performance liquid chromatography; LC-MS/MS, Liquid chromatography with tandem mass spectrometry; T, Testosterone.

^a^At week 24; ^b^Month 2–6; ^c^At month 6; ^d^Month 1–12; ^e^Previously treated children, at all timepoints; ^f^Month 1–48; ^g^All patients, at week 4, E2 <18.36 pmol/L; ^h^Treatment-naïve, at month 6; ^i^All patients, at Week 24; ^j^All patients, at month 60; ^k^All timepoints post-baseline; ^l^At month 1; ^m^From Month 1 through Month 72; ^n^At month 48; ^o^Not reported for all of these n number (E2 ≤ 5 pg/mL); ^p^All patients, at week 4; ^q^After the first year of treatment; ^r^At Month 12; ^s^Treatment-naïve, at month 48; ^t^All patients, during the first 72 weeks; ^u^Treatment-naïve, within 6 months of treatment; ^v^All patients, at month 6; ^w^All patients, at week 48; ^x^Breast Tanner stage stabilized or regressed in 81.8% of females at week 4; ^y^Breast development stabilized or regressed in 91% of girls at month 6; ^z^Breast development stabilized or regressed in 82% of girls at month 6; ^aa^Tanner stage stable or reduced in 90.9%/88.6% of patients between baseline and month 6/12; ^ab^Clinical signs of puberty stabilized or regressed in almost all girls (55/57) at week 48; ^ac^Tanner breast stage at month 60 is similar to baseline in treatment-naive female patients but lower in previously treated female patients.

* It should be noted that International Units (IU) used to express serum concentrations of LH are calibrated based on guidance from the WHO Expert Committee on Biological Standardization. This committee provides a reference preparation of LH, sets the number of IUs contained in that preparation, and specifies a procedure to compare other preparations of the same agent to this preparation. When the supply of reference preparation is depleted, a new version is prepared, sent out, and assays are revalidated. The first LH international standard was issued in 1988, and there have been four subsequent updates. For example, 1 IU of LH was ~0.2 μg LH in version 3 and ~0.3 μg LH in version 5 (29, 30). Therefore, the variability of the definition of one IU over time makes comparisons of results across clinical trials difficult and likely invalid, as efficacy endpoints derived from the use of older versions would need to be validated or recalibrated to match newer versions (29, 30).

** Assay type and lower limit of detection (LLOD) are listed for each trial, but efficacy data are limited by the sensitivity of the assays. For example, Klein et al. found that E2 levels were considerably lower when measured by a research ultra-sensitive bioassay (LLOD = 0.02 pg/mL) in comparison to a radioimmunoassay (31), indicating that ultra-sensitive assays should be used to monitor treatment efficacy in children with CPP. The most sensitive commercially available E2 assays to date are LC-MS/MS.

### Caution with cross-study comparisons

Comparing safety and efficacy data among trials requires caution as they are conducted under widely varying conditions. Important confounding factors include different participant populations, thresholds for hormone levels in defining efficacy, assays and instrumentation, availability of FACTREL^®^ for stimulation tests, trial lengths, routes of injection, and dosages. Differences in demographics and characteristics (age, ethnicity, baseline hormone levels, etc.) of study participants at baseline can also affect results. Different investigators may arrive at different conclusions with respect to subjective assessments. An important additional consideration is that the standards for defining International Units for LH have changed over time, so comparison of results from trials using different versions may not be valid (32). Additionally, pivotal trials were conducted during different decades, during which many factors (e.g., hormone assay sensitivity and instruments) may have changed.

## Overview of currently FDA-approved therapies

### Intramuscular leuprolide acetate

Leuprolide acetate is a synthetic non-apeptide analog of naturally occurring GnRH (13). Intramuscular LA for use in pediatric populations (LUPRON DEPOT-PED^®^) is administered every 28 days [7.5 mg/11.25 mg/15 mg (1 mL)] or every 12 weeks [11.25 mg/30 mg (1.5 mL)] (13). The 1-month formulation received FDA approval in 1993, with dosing based on body weight (13). The 3-month formulations received FDA approval in 2011 with dosing not based on body weight (14).

#### One-month intramuscular leuprolide acetate

In the pivotal trial, investigators performed GnRH stimulation tests using FACTREL^®^ (native GnRH) at a dose of 100 μg IV with blood samples taken at 0, 20, 40, 60, and 90 min post-stimulation (22). Mean peak GnRH-stimulated luteinizing hormone (LH) was suppressed to 0.8 IU/L by week 24. Mean random LH decreased from 2.0 IU/L at baseline to 0.5 IU/L at week 4 in girls and from 2.4 IU/L at baseline to 0.5 IU/L at week 4 in boys. Mean estradiol (E2) in girls decreased from 15.6 pg/mL (57.3 pmol/L) at baseline to <5.0 pg/mL (<18.4 pmol/L) by week 4. Mean testosterone (T) in boys decreased from 199.8 ng/dL (6.9 nmol/L) at baseline to 17.8 ng/dL (0.6 nmol/L) by week 4. Mean ± SD time to first menses after discontinuation of treatment was 1.5 ± 0.5 years (range: 0.5–2.5 years). A post-study survey conducted until girls were 21 years of age reported normal menstrual cycles in 80% of girls and six live births (22). All pregnancy attempts were successful (22).

Long-term data for 1-month IM LA are available. One study collected data on outcomes for 1-month IM LA over 18 years (1991–2009) (23). Mean bone age (BA) was advanced 3 years beyond chronological age (CA) prior to treatment initiation. The mean ratio of change in BA to change in CA was 0.7 after the first year of treatment and remained <0.6 during the next 3 years of treatment (23). Girls who participated in this trial had a mean mid parental height of 163.8 cm and a mean predicted adult height (PAH), based on current height and bone age, of 157.4 cm at baseline (23). Mean attained (near) adult height was 162.5 cm, representing a mean gain of 4.0 cm over initial PAH (23). A separate study evaluating girls treated with 1-month IM LA reported a mean ± SD near-adult height of 162.5 ± 7.4 cm (range: 146.5–176.1 cm), with a mean ± SD change in PAH during treatment of 7.3 ± 6.2 cm (range: −4.4 to 13.6 cm) in children <7 years at treatment start and 5.3 ± 4.6 cm (range: −2.9 to 14.6 cm) in children ≥7 years at treatment start. BA/CA ratio decreased from pretreatment values, averaging 1.5–1.2 at the end of treatment (33).

#### Three-month intramuscular leuprolide acetate

In the pivotal trial, investigators performed GnRHa stimulation tests using SQ injections of LA at a dose of 20 μg/kg (24). Peak GnRHa-stimulated LH (determined at 30- and 60-min post-stimulation) was suppressed to <4 IU/L in 78.4% of participants who received the 11.25-mg dose and 95.2% of participants who received the 30-mg dose from months 2–6. With treatment, almost all participants achieved prepubertal E2 or T concentrations [E2 <20 pg/mL (73.4 pmol/L); T <30 ng/dL (1 nmol/L)] at all visits (93.0 and 100.0% for participants who received 11.25 and 30 mg, respectively). Decreases in BA to CA ratios (BA/CA) at month 6 were observed in 89.7% of participants in the 11.25-mg group and 75.0% of participants in the 30-mg group. A follow-up study over 36 months showed that 3-month leuprolide acetate was associated with an acceptable safety profile and provided maintenance of LH suppression in the majority of children with CPP during the 36 months of the study or until readiness for puberty. 85.3% of participants in the 11.25-mg group and 94.7% of participants in the 30-mg group had LH values <4 IU/L after day 1 at all time points (34).

### Six-month intramuscular triptorelin pamoate

Triptorelin pamoate (TRIPTODUR^®^) is a synthetic decapeptide GnRHa administered every 24 weeks [22.5 mg (2 mL)] that received FDA approval in 2017 (15). In the pivotal trial, GnRH stimulation tests were performed using SQ injections of LA at a dose of 20 μg/kg and peak-stimulated LH was assessed 30 min post-stimulation (26). Peak GnRH-stimulated LH levels of <5 IU/L were achieved in 93.2% (41/44) of participants at month 6 and in 97.7% (43/44) at month 12 (26). A decrease in BA/CA occurred in 63.6% at month 6 and in 95.5% at month 12. Mean ± SD for BA/CA was 1.4 ± 0.2 at 6 months and 1.3 ± 0.2 at 12 months. Exploratory analysis using a lower cut-off showed that 90.9% (40/44) of participants achieved peak GnRH-stimulated LH levels of <4 IU/L at month 6. An additional 9-year-old boy did not maintain peak GnRH-stimulated LH suppression to the lower cut-off (LH of 4.1 IU/L at 6 months).

### Six-month subcutaneous leuprolide acetate

Subcutaneous LA (FENSOLVI^®^) is administered every 6 months (24 weeks) [45 mg (0.375 mL)] and received FDA approval in 2020 (16). In the pivotal trial, GnRHa stimulation tests were performed using SQ injections of LA, either 20 μg/kg or 500 μg (fixed dose), depending on the study site (27). Post-GnRHa-stimulated (30 min post-stimulation) LH <4 IU/L was achieved by 87.1% (54/62) of participants at week 24 and by at least 85.0% at all time points up to the end of the study period (week 48) (27). Mean ± SE post-GnRHa-stimulated LH levels were 3.0 ± 0.8 IU/L at week 24 and 2.3 ± 0.2 IU/L at week 48. In this study, 96.7% (58/60) of girls and 100.0% (2/2) of boys achieved prepubertal E2 and T concentrations [E2 <20 pg/mL (<73.4 pmol/L); T <28.4 ng/dL (<1 nmol/L)], respectively, at week 24. At week 48, 98% of girls achieved E2 <10 pg/mL (<36.7 pmol/L). Of the two boys, one achieved peak LH suppression <4 IU/L and T <28.4 ng/dL at week 48; however, the other boy had above-target peak LH and T levels (27). Mean growth for all children slowed throughout the treatment: from 8.9 ± 1.7 cm/year at week 4 to 5.4 ± 0.5 cm/year at week 24 and 6.0 ± 0.5 cm/year at week 48 (27). Mean BA was advanced by 3.0 years beyond chronological age at screening and was 2.7 years at week 48 (27).

### Twelve-month histrelin acetate implant

Histrelin acetate, a synthetic non-apeptide GnRH analog implant inserted surgically, is available as a 12-month 50-mg dose (SUPPRELIN^®^) that received FDA approval in 2007 (17). In the pivotal trial, investigators performed GnRHa stimulation tests using SQ injections of LA at a dose of 20 μg/kg, and obtained peak-stimulated LH concentrations at 30 and 60 min post-stimulation (35). Peak GnRHa-stimulated LH suppression <4 IU/L was achieved in all treatment-naïve participants and maintained in all pretreated participants through month 12 (28, 35). Peak GnRHa-stimulated LH levels declined throughout treatment with a mean ± SD of 0.8 ± 0.4 IU/L and 0.5± 0.3 IU/L in treatment-naïve and pretreated groups after 1 month, respectively (35). Mean ± SD random LH level was 0.4 ± 0.3 IU/L (36). Estradiol <20 pg/mL (<73.4 pmol/L) was achieved in 100.0% of girls through month 9 and T <30 ng/dL (<1.0 nmol/L) was maintained in all boys previously treated with a standard GnRHa regimen for at least 6 months. Mean ± SD for BA/CA ratio decreased from 1.4 ± 0.2 at baseline to 1.3 ± 0.1 at 12 months. Predicted adult heights were estimated for participants eligible for a long-term extension of the initial trial (28). In girls, PAH increased by 14.6 cm from baseline to month 60. Predicted adult height for the only boy in the extension trial increased by 3.8 cm from baseline to month 60. There is evidence that the implant is effective for much longer than 12 months. Hirsch et al. found that basal and stimulated LH and E2 remained suppressed 15 months after implant insertion (37), and Lewis et al. found equivalent LH suppression when comparing data at 12 and 24 months (38).

## Factors in treatment selection

Different therapies have different active ingredients, drug delivery systems, and routes of administration. Therefore, different doses are required to ensure exposure to effective levels of drug and, consequently, clinical efficacy throughout the dosing period. Drug and administration characteristics, including needle size, injection volume, duration of action, treatment monitoring, and cost, also differ and are important factors in treatment selection.

### Active agent

All GnRHa's used to treat children with CPP downregulate GnRH receptors, reduce LH and FSH release, and suppress ovarian and testicular production of E2 and T, respectively (13, 15–17). Therefore, no significant differences in efficacy should be expected among therapies, provided adequate doses and exposures are delivered throughout the dosing period. One- and 3-month LA received the earliest FDA approval for treatment of children with CPP, followed by the 12-month histrelin acetate implant and 6-month triptorelin pamoate. Children and their caregivers should be aware that post-marketing reports of allergic reactions (anaphylaxis, rash, urticaria, and photosensitivity reactions) (13, 15–17) and convulsions (13, 15, 16) have been observed with GnRHa's (15–17). In April 2022, a warning that idiopathic intracranial hypertension has been reported in pediatric patients receiving GnRHa's was added to drug labels for all therapies discussed in this review (13, 15–17). The new warning advises that patients should be monitored for signs and symptoms, including headache, papilledema, and blurred vision (13, 15–17).

### Formulation

Both 1- and 3-month IM LA formulations use a microsphere delivery technology that embeds the active ingredient in microcapsules made of biodegradable polymers (13, 39). The LA is then released in two phases: a diffusion, or “burst,” phase immediately after injection and a slower bioerosion phase as the polymers degrade (39, 40). Subcutaneous LA is formulated with a polymeric gel delivery system that forms a single solid after injection (41). Consistent with the expected pharmacokinetics of controlled-release formulations, SQ LA is characterized by an initial “burst” release of the active drug followed by a plateau phase (27). Extended-release IM triptorelin pamoate uses a biodegradable microgranule formulation (13, 15, 16). The histrelin acetate implant formulation embeds the active ingredient in a non-biodegradable, diffusion-controlled polymer (17). If children experience therapy-related hypersensitivity, the implant can be removed immediately (42). The varying delivery systems used may require different doses of active molecules to ensure effective exposure to the drug throughout the dosing period.

### Route of administration

The route of administration of medications may also affect selection. Histrelin implants avoid multiple injections over years. Eugster et al. reported that placement and removal of implants is a minor outpatient procedure easily accomplished with local anesthesia (43), often with a child life specialist in attendance to reduce stress (44). Child-reported pain or discomfort after the insertion procedure is less likely when performed under local anesthesia (37). Some institutions use general anesthesia for implant placement, especially for very young children or for children with developmental disabilities. Per FDA guidelines, it is recommended that histrelin acetate implants be removed or re-inserted every year (17). However, a study has demonstrated that a single implant may be effective for at least 2 years, potentially requiring fewer overall office visits and procedures (38). Implant breakage (fracture) and/or difficulty with localization have been reported during removal (45). The risk of implant fracture increases with the length of time the implant is left *in situ*, particularly if this exceeds 2 years (38, 45) with breakage rates of 22–28% during removal (28, 38, 43, 46, 47). There are reports that retained implant pieces may lead to the continued suppression of sex hormones (48), which may be a concern if the child is lost to follow-up (38, 49). A recent case report of a boy treated with the histrelin implant and subsequently lost to follow-up described continuous gonadotropin suppression for 7 years (50).

Injections avoid the minor surgery and anesthesia required for insertion and removal of implants (17, 19). Injection-site pain and erythema have been observed in children who received each of the IM or SQ GnRHa options (16). Sterile abscess formation has been reported following administration of IM LA, triptorelin, and histrelin, with rates ranging from 0.6 to 5% (51–54). The exact cause of sterile abscesses is unknown and hypotheses include an inflammatory reaction to the polymer used in the delivery system and the injection method (53). Children who develop sterile abscesses may have their therapy formulation changed (51, 52). Nafarelin, a rarely used intranasal GnRHa, may be an option in this situation. In some cases of recurrent sterile abscess formation even after changing therapies, treatment may need to be discontinued (51).

Subcutaneous injections with shorter needles may lower the risk of adverse events that are seen with IM injections, such as secondary swelling, hematoma, and rarely, bone or nerve injury (55). Research suggests that the convenience and tolerability of the SQ route will likely be valuable for children (16, 56–58). A review authored by experts from multiple international pediatric endocrinology societies noted that clinical care using GnRHa's has trended toward the use of SQ over IM for long-acting injections, with similar efficacy and much less pain (19).

### Needle size and injection volume

Fear of painful procedures is more common in children than adults (59, 60). Potential strategies to minimize discomfort and anxiety include the use of shorter needles, thinner needles, and smaller injection volumes. Shorter needles may be less intimidating and provoke less fear even if they are thicker (61, 62). Thinner needles may cause less pain (63, 64) and pediatric injections typically use needles with gauges of 22 or above (65). Administration of SQ LA requires a thicker needle (18-gauge) than IM LA (23-gauge) and triptorelin (21-gauge) due to its viscous formulation.

Smaller injection volumes are associated with less pain (66). Pediatric nursing procedures recommend that injection volumes for the IM route not exceed 1.5–2.0 mL, depending on the site of administration (67). Intramuscular LA has volumes of 1.0 or 1.5 mL, triptorelin pamoate is 2.0 mL, and SQ LA is 0.375 mL (13, 15, 16). Volumes of 1.2 mL or higher have been significantly associated with increased pain following injection (68).

Injection reactions may be associated with injection site, injection depth, injection volume, needle length/gauge, administration techniques, etc. Clinicians may opt to use topical or local anesthetics to numb the injection site. Psychological and distraction techniques decrease anxiety prior to and during injection (60).

### Duration of action

More frequent injections required for shorter-acting formulations may contribute to dosing non-adherence. A 7-year retrospective analysis of children who received 1-month IM LA for CPP found that only one quarter of them received subsequent injections within the recommended 28-day administration period, with a mean of 37 days between doses (69). Serum concentrations of the active drug may drop to below therapeutic levels if the drug is administered late, so consistent on-time dosing is important in clinical practice to avoid loss of hormone suppression.

Formulations with duration of action of 6 months or more provide fewer occasions for children to experience fear and anxiety related to healthcare settings and/or interactions with medical professionals (70). However, less frequent contact with the treating physician increases the possibility that important changes in disease progression may not be identified in a timely manner, and it is not yet known how many children receive treatment within the recommended dosing periods, or how quickly an increase in sex hormones occurs if a dose is delayed. Children who have received histrelin acetate implants will benefit from a full year without potential to miss a dose, but they may also fail to return in a timely manner for an office visit to replace or remove the implant (49). Although there is evidence that these implants may be effective for up to 2 years in many children (and even longer in some), it is still important to consider the potential lack of adequate suppression with delayed re-insertion (38). Loss to follow-up could also mean suppression of puberty for longer than intended. With all GnRHa's, it is important to schedule regular follow-up appointments to monitor the degree of hormonal suppression and clinical improvement, and assure continued treatment for as long as is necessary.

There are also educational and economic dimensions to the duration of action of therapies. Scheduling of visits can be challenging and may mean that children and caregivers are required to take time off school or miss work, respectively (37). Home injections may be available for some patients, which will be beneficial for children and their caregivers who live far from their healthcare facility.

Duration of action affects not only child and clinician experience and convenience, but also the flexibility when planning cessation of treatment. Frequent injections with shorter durations of action allow more flexible timing in terms of defining treatment termination. It is possible to switch between agents at any time to facilitate this, with the new agent always administered the day the previous treatment was due.

### Monitoring during treatment

Clinical signs of puberty, growth rate, rate of bone maturation, levels of LH and sex steroids, and estimates of change in PAH are commonly used to assess efficacy of CPP therapies. Response to treatment varies between individuals, with some studies finding an association between lower LH levels, less BA advancement and greater increases in PAH (71). However, small differences in LH suppression may not be clinically relevant if other measures of treatment efficacy indicate an adequate response. It is important to assess E2/T concentrations and clinical outcomes in addition to stimulated LH concentrations, as it is these sex steroids that directly cause the advances in pubertal maturation, pubertal growth spurts, and BA maturation (72). To date, random hormone concentrations alone have not proven adequate when assessing treatment benefit with GnRHa's, so assessment of all clinical and laboratory parameters, in combination with rate of BA maturation, is essential to interpret treatment response. Studies of attainment of near-predicted adult height may determine whether levels of LH suppression result in optimal treatment outcomes. Most studies suggest that a GnRH-stimulated peak LH <4 IU/L is useful, provided that physical signs of pubertal maturation, height velocity, and rate of BA progression are also consistent with a suppressed HPG axis (73). A random LH level <0.6 IU/L *may* also indicate adequate suppression (73), but higher levels are not necessarily indicative of treatment failure (36). When determining a random LH threshold indicating adequate suppression, it is important to consider that random LH is higher during the night than during the day (74). Therefore, the threshold may vary depending on when the measurements were taken. Earlier studies regarding determining age for treatment cessation suggest that optimal height gains are achieved when treatment is stopped at a BA of ~12 years in girls (75, 76). However, more recent analyses stress the importance of individualizing the decision of when to stop therapy based on multiple variables, including rate of bone age progression, rate of linear growth, and changes in PAH (33). Some girls with BA >12 years have significant height potential if treatment is continued longer.

### Cost

Treatment for CPP can be costly, so it is a key consideration for children and their families. In the year following treatment initiation, children with CPP spent six- to 12-times more on healthcare costs compared with matched controls (patients without CPP), largely due to spending on outpatient services and outpatient pharmacy claims (77). Third-party coverage typically determines the cost to families or caregivers, hence it is important for clinicians to work with caregivers to find a treatment option suitable for their financial considerations in addition to the clinical and medical considerations.

## Discussion

Some hypotheses regarding CPP and appropriate treatment require further study. For example, it has been suggested that prolonged GnRHa administration may negatively impact future reproductive function, body composition [as measured by body mass index (BMI) and/or lean and fat body mass] (78), and/or bone health (8, 79). However, these claims are controversial and are not backed by existing clinical data (19). A recent review by an international consortium reported a lack of evidence that GnRHa treatment impairs adult reproductive function or fertility (19), and a separate study found that 84.4% of pregnancies in women previously treated with GnRHa's for CPP occurred within 1 year of trying to conceive, suggesting that fertility in adulthood was not negatively impacted (80). Data on long-term outcomes in males are limited, but there appear to be no differences in sperm count or gonadal function between males previously treated with GnRHa's for CPP and those who were not (81, 82). A proposed link between the use of GnRHa's and increased risk of obesity is also unsubstantiated. Girls with CPP have higher overall BMI at the time of diagnosis than those with normally-timed pubertal onset (8), but GnRHa treatment does not appear to influence progression toward obesity during adolescence or adulthood or impact body composition (19, 83). Data suggest that, while children treated with GnRHa's have a diminished bone accrual during treatment, bone mineral density (BMD) will likely be within the normal range by late adolescence well after treatment is concluded (19). In a study comparing healthy children to children with CPP treated with GnRHa's, no significant difference was seen in BMD. Additionally, no significant difference in BMD was detected between pre- and post-treatment in children with CPP (84). Some literature has suggested that early menarche resulting from untreated CPP may increase the risk of estrogen-sensitive breast and reproductive-tract malignancies (4, 85). However, one study of 142 women previously diagnosed with CPP found no significant difference in the rate of malignancies between women with CPP and healthy controls or between women formerly diagnosed with CPP who were treated and those who were not (86). These findings are consistent with previous comparisons of women with breast cancer to those without. One study of 425,055 women found that breast cancer risk increased by a factor of 1.05 (95% CI 1.044–1.057, *p* < 0.001) for each year younger at menarche (87). Another study reported a hazard ratio of 1.10 (95% CI 1.01–1.20) for early age at menarche (<12 years) and increased breast cancer risk (88). Data on the impact of untreated, or ineffectively treated, children with CPP on QOL and psychosocial functioning have been inconclusive (89, 90). Additional studies to evaluate possible associations between children with untreated CPP and cancer risk, as well as the effects of GnRHa therapy on emotional and behavioral function of children with CPP, have been recommended (8, 79). As one of the primary goals of CPP treatment is to improve the child's likelihood of achieving the genetic target height range, longer-term studies would provide valuable data on how each of the newer agents impacts adult height.

Although there are few direct comparison studies and evaluation across studies requires caution, published data suggest similar safety and efficacy over the first year of treatment among all FDA-approved therapies for CPP. Intramuscular LA (1- and 3-month formulations) are established products with long-term safety and efficacy data and a comparatively shorter duration of action that may allow for greater flexibility in dosing and termination of treatment (17). Intramuscular triptorelin pamoate (6-month) offers a long duration of treatment and the advantage of fewer injections, but there are limited data on long-term efficacy and outcome. Six-month SQ LA delivers a molecule with a long history of use *via* a unique technology, addressing some treatment preferences including small volume, SQ injection and long duration of action, but long-term efficacy and safety data are not available (14). The 12-month (or longer) SQ histrelin acetate implant offers the longest duration of action with evidence of appropriate long-term clinical and biochemical suppression, but administration requires minor surgery with local or general anesthesia, and implant fracture during removal is possible (15). These factors should be balanced against less frequent visits for monitoring and medication administration. It is possible to switch between agents at any time during a course of therapy and this also allows for flexibility when planning treatment duration and cessation. The current landscape of available GnRHa's for the treatment of children with CPP provides options with varying features, benefits, and risks, allowing physicians and caregivers to select the most appropriate therapy based on the specific needs and concerns of the child and the caregiver.

## Author contributions

All authors contributed to the conception, drafting, and revision of this manuscript. All authors approved the final version for publication and agreed to be accountable for all aspects of the manuscript.

## Funding

Writing support provided by Xelay Acumen Group and funded by Tolmar Pharmaceuticals, Inc.

## Conflict of interest

Author AR is a consultant for Tolmar Pharmaceuticals, Inc. Author JP is a consultant for Tolmar Pharmaceuticals Inc., and is listed on the speaker's bureau for AbbVie. Author MG is a clinical trial site consultant for Endo Pharmaceuticals Inc., served as a member of a Data Safety Monitoring Board for the FENSOLVI^®^ trial for Tolmar Pharmaceuticals Inc., and received royalties from McGraw-Hill and UpToDate. Author LS is a consultant/advisor for Tolmar Pharmaceuticals Inc., served as a member of a Data Safety Monitoring Board for the FENSOLVI^®^ trial for Tolmar Pharmaceuticals Inc., and is a consultant/advisor for Endo Pharmaceuticals Inc., Myovant Sciences, and Enteris Biosciences. Author PK served as a Chair of a Data Safety Monitoring Board for the FENSOLVI^®^ trial for Tolmar Pharmaceuticals Inc. Author NM served as site PI for clinical trials sponsored by Tolmar Pharmaceuticals Inc., and received grant support from AbbVie. Author PZ served as a member of a Data Safety Monitoring Board for the FENSOLVI^®^ trial for Tolmar Pharmaceuticals Inc. Author EE served as site PI for clinical trials sponsored by Tolmar Pharmaceuticals Inc., and AbbVie. Author KK is a consultant for Tolmar Pharmaceuticals Inc., AbbVie, and Arbor.

## Publisher's note

All claims expressed in this article are solely those of the authors and do not necessarily represent those of their affiliated organizations, or those of the publisher, the editors and the reviewers. Any product that may be evaluated in this article, or claim that may be made by its manufacturer, is not guaranteed or endorsed by the publisher.
